# Advances in the Synthesis of Fused 1,2,3-Triazoles via a MCR-Intramolecular Azide-Alkyne Cycloaddition Approach

**DOI:** 10.3390/molecules28010308

**Published:** 2022-12-30

**Authors:** Rahul P, Joice Thomas, Wim Dehaen, Jubi John

**Affiliations:** 1 Chemical Sciences and Technology Division, CSIR-National Institute for Interdisciplinary Science and Technology (CSIR-NIIST), Thiruvananthapuram 695019, India; 2 Academy of Scientific and Innovative Research (AcSIR), Ghaziabad 201002, India; 3 The Bridge@USC and Loker Hydrocarbon Research Institute, University of Southern California, Los Angeles, CA 90089, USA; 4 Molecular Design and Synthesis, Department of Chemistry, KU Leuven, Celestijnenlaan 200F, 3001 Leuven, Belgium

**Keywords:** 1,2,3-triazole, multicomponent reaction, intramolecular azide-alkyne cycloaddition, dipolar cycloaddition

## Abstract

The present review narrates several reports which deal with the synthesis of fused 1,2,3-triazole containing scaffolds following a sequential multicomponent reaction (MCR)—intramolecular azide-alkyne cycloaddition (IAAC) approach. The reviewed reactions were cleverly designed so as to incorporate azide and alkyne functionalities in the MCR product which was then subjected to IAAC. The review is divided into two sections based on the number of components in the multicomponent reaction. We have aimed at a critical discussion and also have highlighted either advantages or disadvantages of each methodology.

## 1. Introduction

**Scheme 1:**
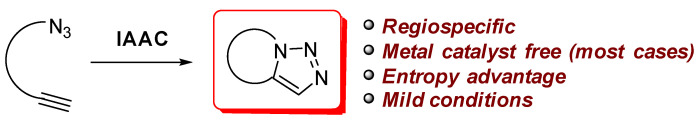
Intramolecular azide-alkyne cycloaddition towards fused 1,2,3-triazoles.

During the past three decades, after the introduction of metal catalysed azide-alkyne cycloaddition, motifs containing 1,2,3-triazole rings have found various applications in areas of synthetic, medicinal and material chemistry [1,2,3,4,5,6,7,8,9]. For example, 1,2,3-triazoles were extensively studied for their biological properties due to amide-triazole bioequivalence [4,10,11,12,13,14]. Fused triazoles, which can be accessed by intramolecular azide-alkyne cycloaddition (IAAC) reactions (Scheme 1), are also found to be very useful moieties, especially in medicinal chemistry [15,16,17].

**Figure 1:**
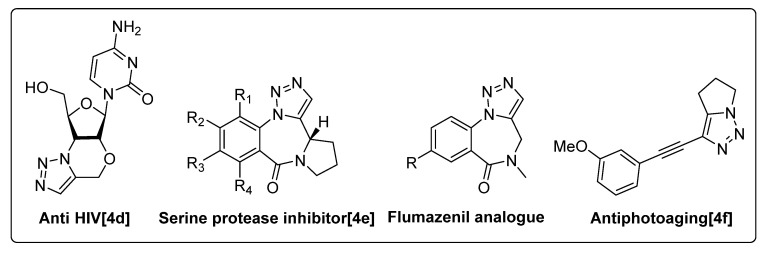
Bioactive moieties containing a fused 1,2,3-triazole motif.

Intramolecular azide-alkyne cycloaddition reactions have some advantages over the intermolecular version including regioselectivity, avoidance of a metal catalyst (in most cases) and mild conditions [15,16,17]. These highlights of IAAC can be attributed to the simultaneous presence of alkyne and azide functionalities in a single reactant and the resulting entropy advantage provided by the intramolecularity. IAAC reactions are being utilized extensively for the synthesis of medium and large ring (carbocycle and heterocycle) fused triazoles. The bio-active molecules which are shown below have been synthesized via a key intramolecular azide-alkyne cycloaddition step (Figure 1) [4,18,19,20,21,22,23]. 

**Scheme 2:**
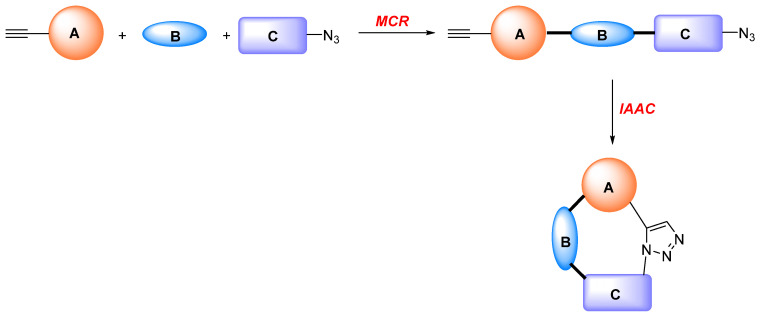
Sequential MCR-IAAC towards fused 1,2,3-triazoles.

Multicomponent reactions (MCRs) are defined as one pot reactions in which three or more different substrates react in a well-defined and orthogonal manner to form a single compound retaining structure or substructure of all starting materials [24,25,26,27]. MCRs allow the formation of several chemical bonds in a single synthetic step leading to densely functionalized linear structures or carbocycles/heterocycles containing several crucial functionalities. Organic chemists have designed MCRs which result in scaffolds containing alkynyl as well as azide moieties in close proximity. Such intermediates may then undergo an intramolecular azide-alkyne Huisgen cycloaddition reaction to provide triazole-annulated poly-heterocycles with complete regioselectivity. The entropic advantage allows this process to be an uncatalysed Huisgen reaction rather than a metal-catalysed reaction, e.g., of the CuAAC (copper catalysed azide-alkyne cycloaddition) (“click”) type, while also favouring the 1,5-disubstituted regiochemistry through proximity effect. Thus, the tandem (or sequential) process of MCR/IAAC has emerged as a synthetic tool to develop highly functionalized fused 1,2,3-triazoles (Scheme 2). 

The purpose of the present feature article is to review different methodologies that are available for the synthesis of fused 1,2,3-triazole heterocycles by following a sequential MCR-IAAC approach. Our analysis is organized based on the number of components involved in the MCR. Thus, the first section deals with different methodologies involving a 3-CR (three-component reaction) followed by IAAC. The second section describes approaches incorporating a sequential 4-CR (four-component reaction) and IAAC towards fused 1,2,3-triazole moieties. In the final section, we have provided future perspectives of the idea discussed in this review.

## 2. Synthesis of Fused 1,2,3-Triazole Moieties via Sequential 3-CR-IAAC Approach

**Scheme 3:**
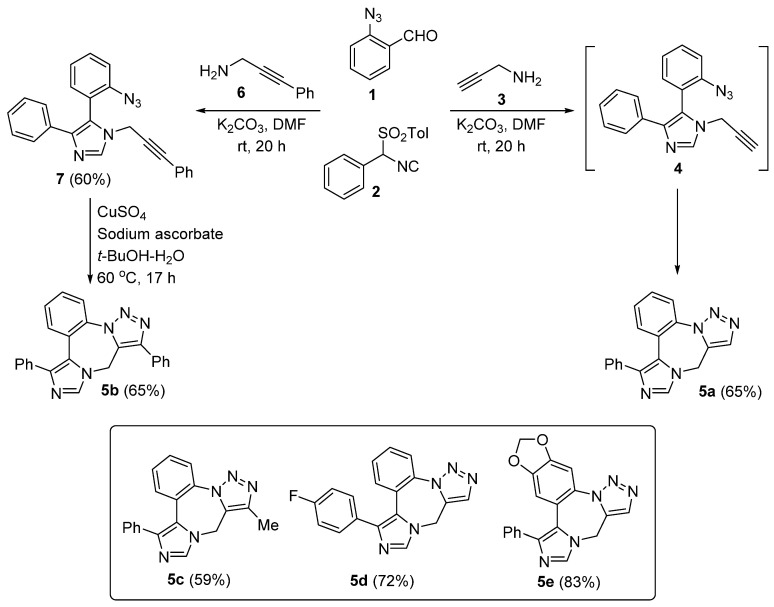
Synthesis of fused triazole-imidazole derivatives.

Gracias and co-workers introduced a facile approach towards the synthesis of fused triazole-imidazole derivatives via sequential van Leusen/IAAC reactions [28]. When a terminal alkyne **3** was used for a van Leusen reaction along with *o*-azido benzaldehyde **1** and the tosylmethylisocyanide **2**, instead of the intermediate imidazole **4**, fused triazole product **5a** was isolated (Scheme 3). When a non-terminal alkyne **6** was used, the van Leusen imidazole **7** was isolated and then subjected to CuSO_4_ catalysed IAAC reaction to afford the fused triazole-imidazole derivative **5b** in 65% yield. However, this is probably not a CuAAC reaction since the accepted mechanism involves monosubstituted alkynes. The details of the role of Cu in this process are lacking in the report. The steric effect due to the presence of the phenyl moiety at the terminal position of the alkyne in **7** might be the reason for the failure of IAAC without the Cu-catalyst. 

**Scheme 4:**
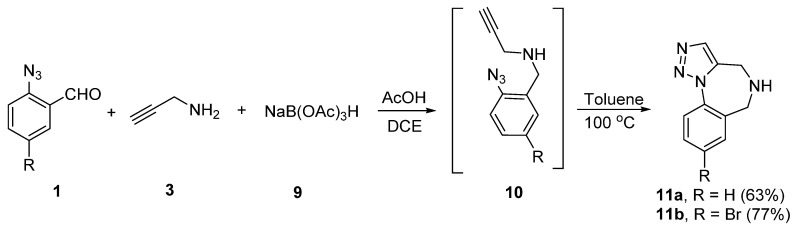
Synthesis of triazolobenzodiazepine by 3-CR.

Martin’s group has worked extensively on the synthesis of triazole-fused scaffolds by sequencing MCRs with IAAC. In 2011, a report appeared on a three component approach towards 1,2,3-triazole-fused 1,4-benzodiazepines [29]. When **1** was treated with propargyl amine **3** and a reducing agent, NaB(OAc)_3_H **9**, the reaction afforded the azido alkyne intermediate **10** which was then subjected to IAAC to afford the fused triazole **11** (Scheme 4).

**Scheme 5:**
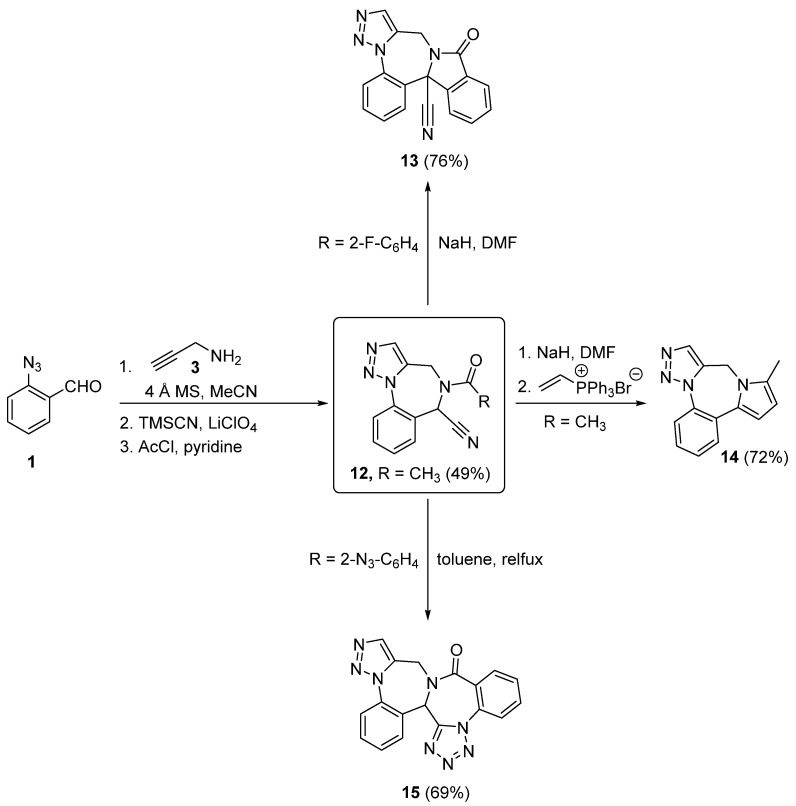
Synthesis of triazolobenzodiazepine with one-pot 4-CR and its derivatization.

In the same report, a simple route was given, leading to a cyano-substituted, triazole-fused 1,4-benzodiazepine **12** starting from 2-azidobenzaldehyde **1a** by combining a Strecker reaction, acylation and IAAC (Scheme 5) [29]. Different acyl moieties were installed on the *N*-4 of the 1,4-benzodiazepine core which was further utilized for synthetic modifications for accessing different interesting fused polyheterocyclic scaffolds **13** to **15**.

**Scheme 6:**
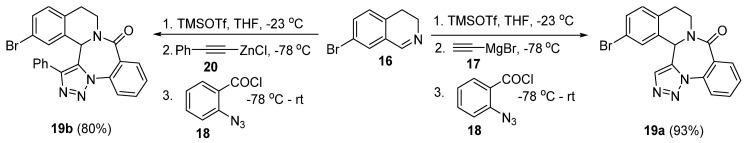
Synthesis of 1,5-benzodiazepin-2-one-fused triazole from dihydroisoquinoline.

Another route towards 1,5-benzodiazepin-2-one fused triazole incorporating an isoquinoline ring was published by the same group starting from 7-bromodihydroisoquinoline **16** [30]. Initially, **16** was allowed to react with ethynylmagnesium bromide **17** in the presence of TMSOTf to afford the intermediate adduct which was then trapped with *o*-azidobenzoyl chloride **18**. The amide formed underwent intramolecular dipolar cycloaddition upon warming to room temperature to afford the triazole 1,5-benzodiazepin-2-one **19a** in 93% yield (Scheme 6). When phenyl zinc-acetylide was used instead of the Grignard reagent, the corresponding triazole-fused benzodiazepinone **19b** was obtained in 80% yield. The aryl bromide functionality was then subjected to Buchwald–Hartwig amination or Suzuki coupling towards further examples of substituted triazolo 1,5-benzodiazepin-2-one-fused isoquinolines. 

**Scheme 7:**
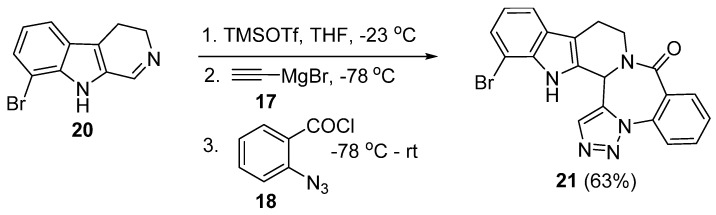
Synthesis of 1,5-benzodiazepin-2-one-fused triazole from dihydrocarboline.

By starting from dihydro-*β*-carbolines and by following the same strategy as shown above, Martin and co-workers extended the library of triazolo 1,5-benzodiazepin-2-ones [31]. Dihydrocarboline **20** was treated with ethynyl magnesium bromide **17** in the presence of TMSOTf which was followed by the reaction of the intermediate adduct with *o*-azidobenzoyl chloride **18** to afford the triazole-fused benzodiazepinone **21** in 63% yield (Scheme 7). 

**Scheme 8:**
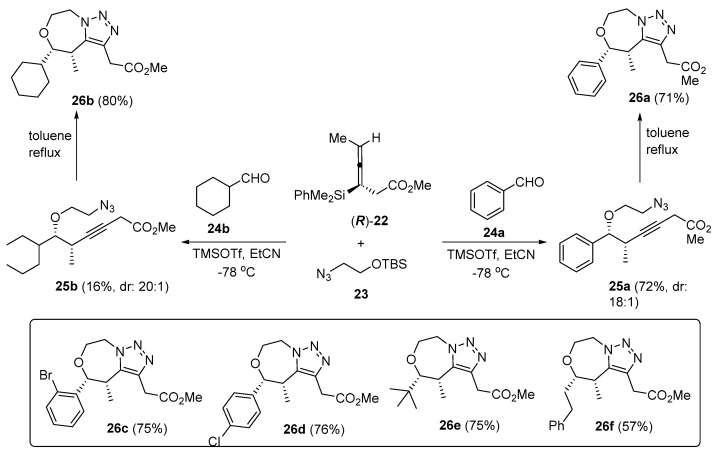
Synthesis of oxepane-fused triazoles.

An interesting route to oxepane-fused triazoles was reported by Panek et al. [32]. The synthetic route involved a three-component coupling of an enantioenriched allenyl silane (*R*)-**22** with an aldehyde **24** and a silyl-protected 2-azido ethanol **23** followed by IAAC. The three-component coupling was done in the presence of TMSOTf in propionitrile at -78 °C to afford the *syn*-propargylic ether with azide and alkyne functionalities positioned suitably for the intramolecular dipolar cycloaddition. Refluxing the product in toluene afforded the corresponding oxepane-fused triazole **26** in good yield. In this case, disubstituted alkynes reacted with azides without the need for metal catalysis (Scheme 8). 

**Scheme 9:**
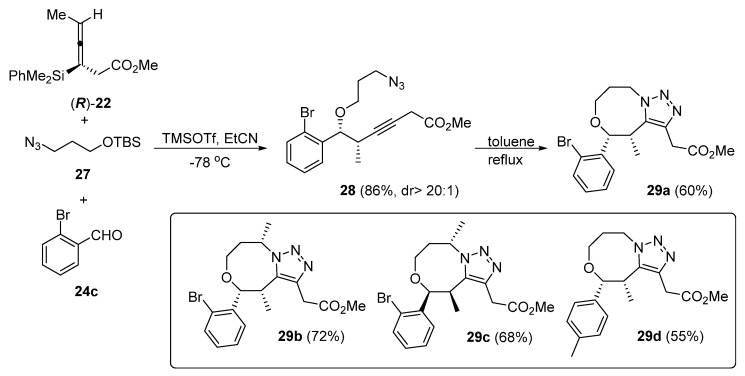
Synthesis of triazolo-fused 1,4-oxazecine derivatives.

The 3CR was found to be general for various aromatic aldehydes, but a lower yield was observed with straight chain aliphatic aldehydes [32]. By substituting TBS-protected azido ethanol **23** with azido propanol **27**, eight-membered oxygen heterocycle-fused triazole **29** could be synthesized in good yields (Scheme 9).

**Scheme 10:**
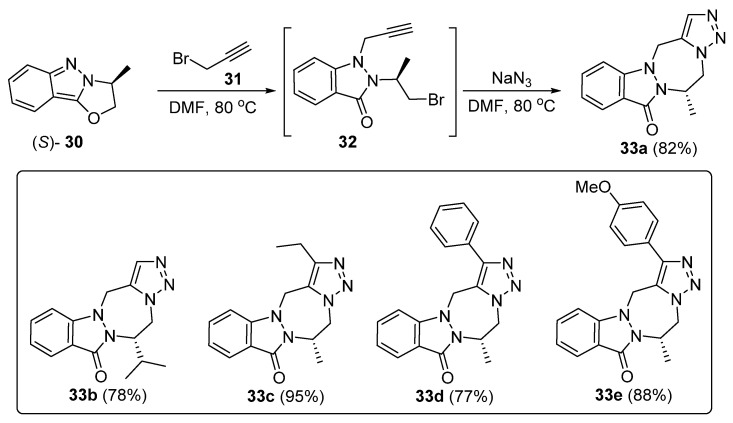
Synthesis of triazolotriazepinoindazolones.

A facile one-pot three component protocol towards triazolotriazepinoindazolones was reported by Kurth and co-workers [33]. The synthesis occurred in two stages and started from oxazolino-2*H*-indazole (*S*)-**30**, which was treated with propargyl bromide **31** at 80 °C for 18 h affording the intermediate **32**. To the same reaction mixture, NaN_3_ was added and heated at 80 °C for 16 h to afford the triazole-fused compound **33a** in 82% yield (Scheme 10). This one-pot, three-step protocol was found to be general for both terminal and internal alkynes and also could accommodate substituents at C3 of the propargyl bromide moiety. 

**Scheme 11:**
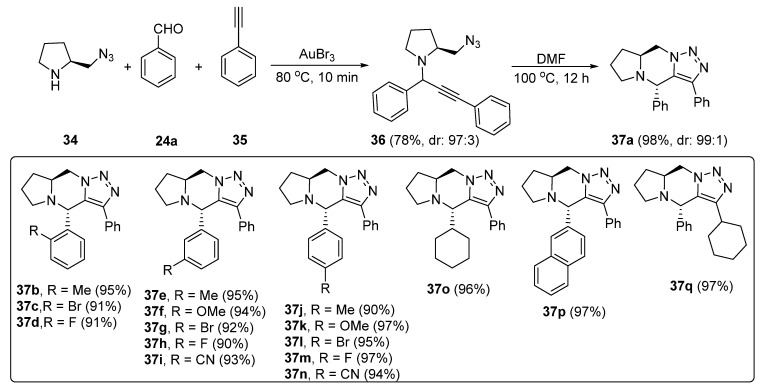
Synthesis of hexahydropyrrolo-triazolopyrazines.

Chen et al. reported an easy route towards triazole-fused pyrazines via A^3^ coupling–intramolecular AAC strategy [34]. The assembly of the crucial intermediate azido-alkyne **36** was realized through the AuBr_3_-catalysed, three-component coupling of L-proline-derived azide **34,** benzaldehyde and substituted acetylene **35**. The compound **36** on heating at 100 °C in DMF afforded the hexahydropyrrolo-triazolopyrazine **37a** in 98% yield and excellent diastereoselectivity (>99%) after 12 h (Scheme 11). It was found that the electronic properties of the substituents of the aromatic ring of the aldehyde did not influence the yield or the selectivity of the A^3^ coupling product **36**. Aliphatic aldehydes were also compatible with the reaction conditions leading to excellent yields of the azido-alkyne. Substituting phenylacetylene with 1-hexyne also did not have an effect on the yield or diastereoselectivity of the reaction. The intramolecular dipolar cycloaddition proceeded smoothly in all cases to afford the fused triazoles in excellent yields. 

**Scheme 12:**
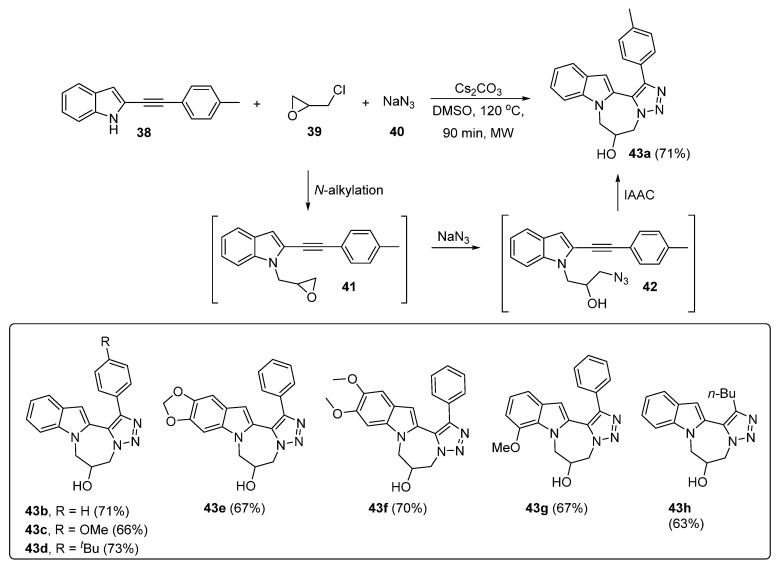
Synthesis of indolodiazepinotriazoles.

An interesting route towards indolodiazepinotriazoles via a microwave-assisted, three-component reaction was reported by Kundu and co-workers [35]. When 2-(4-methylphenylethynyl)-1*H*-indole **38** was treated with epichlorohydrin **39** and NaN_3_ in DMSO at 120 MW, after 90 min, the reaction afforded 71% of the fused triazole **43a**. After 10 min of the reaction, the N-alkylated compound **41** was detected in 80% yield, and after 30 min, the azido-alkyne intermediate **42** was found in 45% along with 20% of compound **41** and 10% of the indolodiazepinotriazoles **43a**. Detection of these intermediates confirms the pathway of the reaction as *N*-alkylation followed by epoxide rings opening from azide moiety and intramolecular azide-alkyne cycloaddition (Scheme 12). The scope of the reaction was well studied, and it was found that the substitution on the phenyl ring of indole did not have any influence on the yield. The end group on the acetylene had a slight influence on the yield as aromatic groups favoured the reactions while the fused triazoles were obtained in lower yield when aliphatic/trimethylsilyl moieties were used. 

**Scheme 13:**
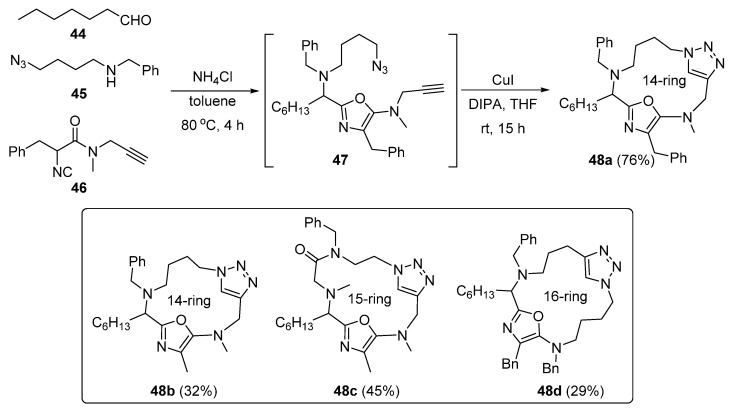
Synthesis of fused triazole-macrocycles.

The first report on the synthesis of macrocycle-fused triazole by following a sequential MCR-IAAC approach came from Zhu and co-workers [36]. The methodology involved the synthesis of 5-aminooxazole intermediate **47** with suitably placed azide and alkyne functionalities, by heating a toluene solution of heptanal **44**, ω-azido amine **45** and isocyanoacetamide **46** in the presence of ammonium chloride. After filtration of the inorganic impurities, the intermediate **47** was subjected to IAAC in the presence of CuI and DIPA which furnished the macrocycle-fused triazole **48** in 76% yield (Scheme 13). The larger 14-membered ring allows the formation of a 1,4-disubstituted triazole, and, therefore, it is advantageous to work under CuAAC-type reaction conditions. By changing the amount of methylene spacers in the starting materials, also, 15- (**48c**) and 16- (**48d**) membered rings could be prepared. 

**Figure 2:**
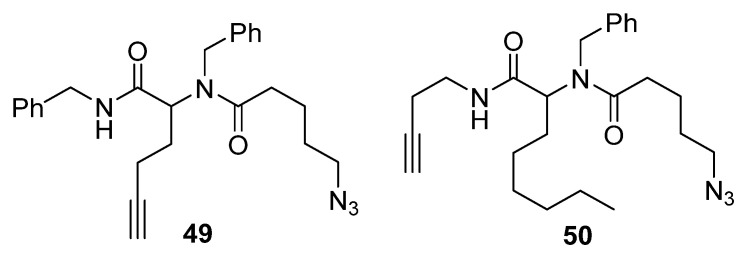
Ugi dipeptide products that failed to undergo IAAC.

It was found that the oxazole moiety provides a conformational preorganization effect during IAAC towards macrocycle formation [36]. This was proven by subjecting dipeptide Ugi products **49** and **50** to various IAAC conditions which failed to furnish macrocycles (Figure 2).

## 3. Synthesis of Fused 1,2,3-Triazole Moieties via Sequential 4-CR-IAAC Approach 

**Scheme 14:**
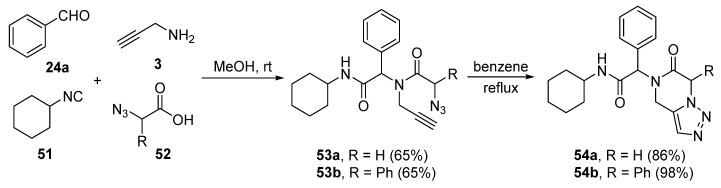
Synthesis of fused dihydrotriazolopyrazinone.

The first report on the synthesis of heterocycle-fused triazole with a sequential multicomponent-IAAC strategy came from the group of Zanze in 2004 [37]. The approach combined the Ugi reaction with intramolecular azide-alkyne cycloaddition to synthesize a variety of six- and seven-membered, ring-fused triazoles in good yields. The starting materials for the Ugi reaction were cleverly functionalized so as to tune the reaction outcome. The intermediate Ugi product **53a** upon intramolecular azide-alkyne cycloaddition afforded fused dihydrotriazolopyrazinone **54b** in 98% yield (Scheme 14). 

**Scheme 15:**
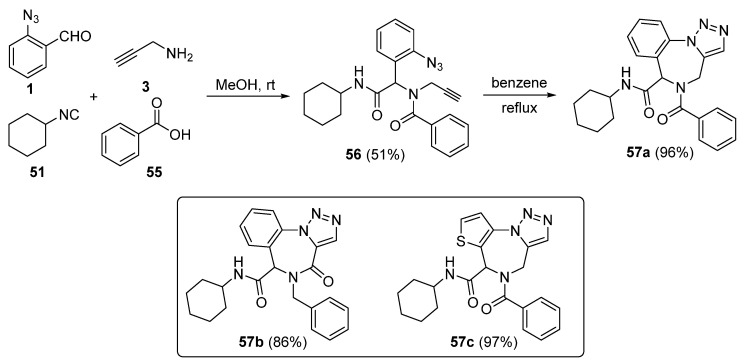
Synthesis of triazolobenzodiazepine.

Triazolobenzodiazepine **57a** was obtained after intramolecular AAC from the Ugi adduct **56**, which was obtained from the multicomponent reaction that involved ortho-azido benzaldehyde **1**, propoargyl amine **3**, cyclohexylisocyanide **51** and benzoic acid **55** (Scheme 15) [37].

**Scheme 16:**
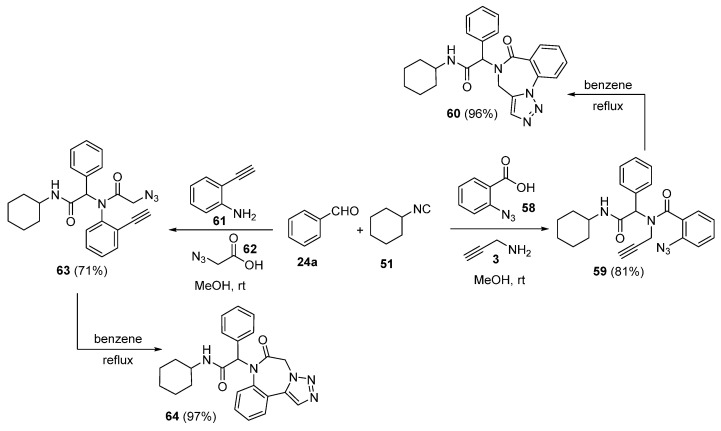
Synthesis of triazolobenzodiazepine.

Different triazolobenzodiazepines were accessed by cleverly installing functionalities on the components of the Ugi reaction [37]. The authors have shown that by combining two different functionalities in one starting material, different triazolo-fused heterocycles could be accessed. In one instance, *ortho*-azidobenzoic acid **58** was used to synthesize the triazolobenzodiazepine **60**, and in another, *ortho*-aminoethynyl benzene **61** was utilized to generate the triazolo fused benzodiazepine **64** (Scheme 16).

**Scheme 17:**
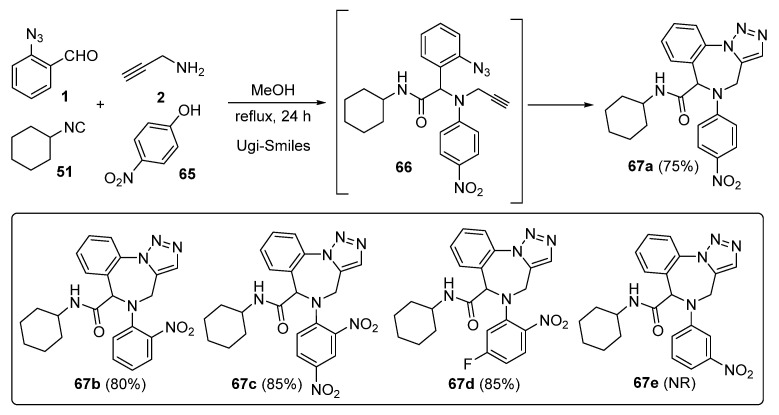
Synthesis of triazolo -used 1,4-benzodiazepines via Ugi–Smiles reaction.

A similar approach towards triazole-fused benzodiazepines utilizing an Ugi-Smiles type reaction was reported by the Shafiee group [38]. Instead of the carboxylic acid used in the above-mentioned strategy, they used different *ortho-* and *para*-substituted nitrophenols. Triazolo-1,4-benzodiazepine **67a** was obtained in excellent yield when a mixture of 2-azidobenzaldehyde **1**, propargyl amine **2**, cyclohexylisocyanide **51** and *p*-nitrophenol **65** was heated in MeOH to reflux for 24 h (Scheme 17). Another observation was that unsubstituted phenols and *m*-nitrophenol did not react under the reaction conditions, due to the lack of sufficient acidity and reactivity. 

**Scheme 18:**
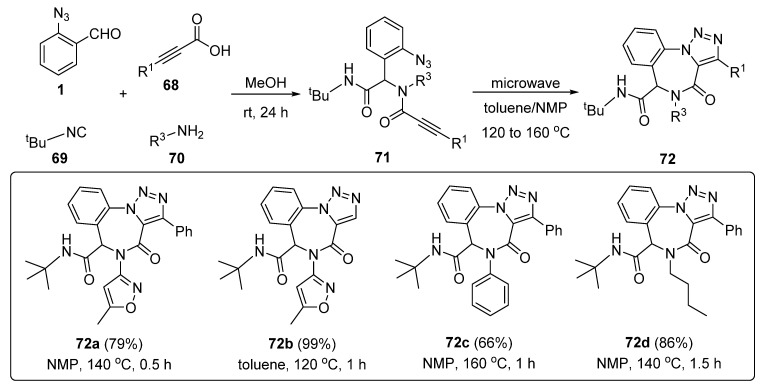
Synthesis of triazolo-fused benzodiazepinone derivatives.

Recently, Chebanov and co-workers reported a microwave-assisted synthesis of 1,2,3-triazolobenzodiazepinones **72** via a sequential Ugi-MCR and IAAC route [39]. The MCR was performed with *o*-azido benzaldehyde **1**, propargylic acid **68**, isocyanide **69** and amine **70** to generate the Ugi adduct **71** with both azide and alkyne moieties suitably placed for the intramolecular dipolar cycloaddition (Scheme 18). The authors performed the IAAC in two ways: First, one was via conventional route wherein the Ugi adduct **71** was refluxed in benzene for 8 h to obtain the 1,2,3-triazolobenzodiazepinone in quantitative yield. Secondly, the Ugi adduct **71** was kept under microwave irradiation at 120 °C for an hour which also resulted in excellent yield of the product. In this work, the authors used both terminal and non-terminal alkynes for IAAC. Typically, with non-terminal alkynes, a catalytic dipolar cycloaddition is performed; in this line, the authors tried several reactions with Rh-catalyst, and all of them failed to afford the fused triazole. All Ugi adducts with terminal and disubstituted alkyne functionalities were subjected to microwave irradiation to effect IAAC for accessing 1,2,3-triazolobenzodiazepinones. 

**Scheme 19:**
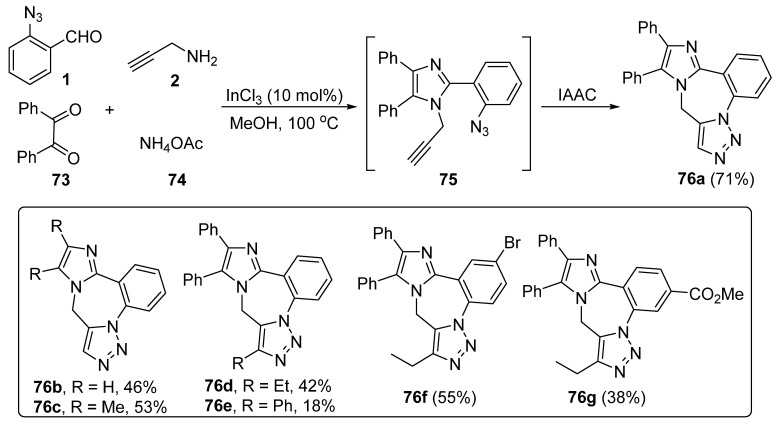
Synthesis of fused triazole-imidazole derivatives.

In 2013, another multicomponent approach towards imidazo-triazolobenzodiazepines was published by Kurth et al. [40]. When benzil **73** was treated with 2-azidobenzaldehyde **1**, propargylamine **2** and ammonium acetate in the presence of InCl_3_ at 100 °C in MeOH, the product imidazo-triazolobenzodiazepine **76a** was obtained in 71% yield via the substituted imidazole intermediate **75** (Scheme 19). The scope of the multicomponent reaction was well studied, and it was found that electronic effects of substituents on each component had an influence on the reaction yield. For example, when phenyl groups on benzil were substituted by hydrogen, the yield decreased to 46%. Also, electron-donating or electron-withdrawing substituents on the aryl group of benzil lowered the yield. In the case of 2-azidobenzaldehyde, the presence of electron-donating moieties had a positive influence on the yield. A light decrease in yield was observed when propargylamine was substituted with internal alkynyl amines.

**Scheme 20:**
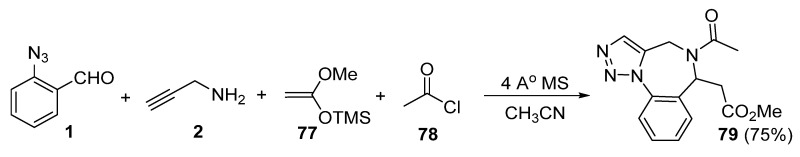
Synthesis of triazolobenzodiazepine by 4-CR.

Martin and co-workers developed a methodology based on 4-CR to prepare various multifunctional heterocyclic systems [41]. In one such attempt, the imine formed from the condensation of 2-azidobenzaldehyde **1** and propargylamine **2** was treated with ketene acetal **77** and acetyl chloride **78** to furnish triazolobenzodiazepine **79** in 75% yield (Scheme 20).

**Scheme 21:**
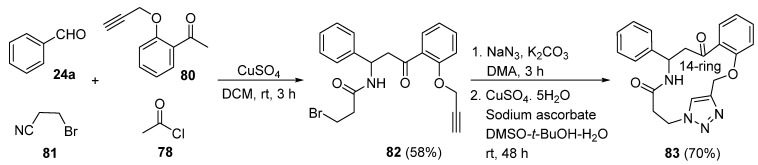
Synthesis of fused peptidotriazole macrocycles.

Another report on the synthesis of macrocyclic peptidotriazoles came from Bahulayan et al. [42]. The methodology involved the preparation of the bromo alkyne intermediate **82** through a CuSO_4_-catalysed MCR of benzaldehyde **24a**, propargylated acetophenone **80**, bromo propionitrile **81** and acetylchloride. The compound **82** was then subjected to azidation reaction with NaN_3_ followed by copper-catalysed intramolecular azide-alkyne cycloaddition (CuAAC) to afford the triazole-fused, 14-membered macrocycle **83** in 70% overall yield (Scheme 21).

**Scheme 22:**
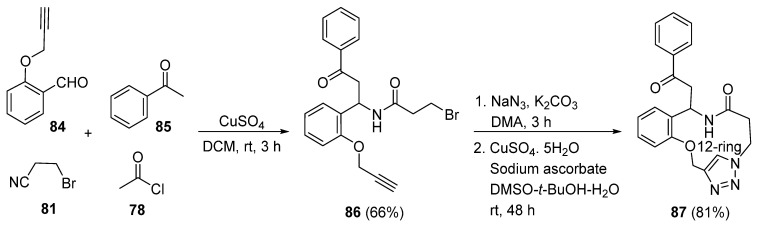
Synthesis of fused peptidotriazole macrocycles.

Similarly, the 12-membered, triazole-fused macrocycle **87** was synthesized by following the same methodology as shown above but by starting from propargylated aldehyde **84** instead of propagylated acetophenone **80** [42]. The intramolecular CuAAC cycloaddition of **86** afforded the macrocycle **87** in 81% overall yield (Scheme 22).

**Scheme 23:**
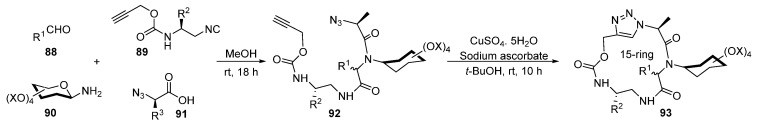
Synthesis of triazole-linked cyclic glycopeptidomimetics.

In 2012, Sureshbabu and team reported the use of a sequential MCR-IAAC strategy for the synthesis of triazole-linked cyclic glycopeptidomimetics (Scheme 23) [43]. The strategy involved the use of simple aldehydes **88**, poc-amino alkyl isonitriles **89** (poc - propargyloxycarbonyl), sugar-1-amines **90** andazido acids **91** as substrates. Initially, these four components were subjected to Ugi reaction conditions to generate the linear Ugi adduct **92** which has the azide and alkyne functionalities appropriately placed for azide-alkyne cycloaddition. The Ugi adduct was then subjected to Cu-catalysed azide-alkyne cycloaddition conditions to generate 15-membered triazole-linked cyclic glycopeptidomimetics **93**. The generality of this methodology was tested by changing the isonitriles **89** and α-azido acids **91**, both of which were synthesized from the corresponding amino acids. Changes were also brought in aldehydes **88** and the amino sugar moieties **90**. In this piece of work, the authors utilized the Poc-group not only as a protecting group for the amine functionality but also as the alkyne component required for the cycloaddition of compound **92**. The authors have pointed out that they have observed the formation of the triazole-linked cyclic glycopeptidomimetics **93** as the major product due to the lesser ring strain for the IAAC, and the dimer was formed only in minor proportions. 

**Scheme 24:**
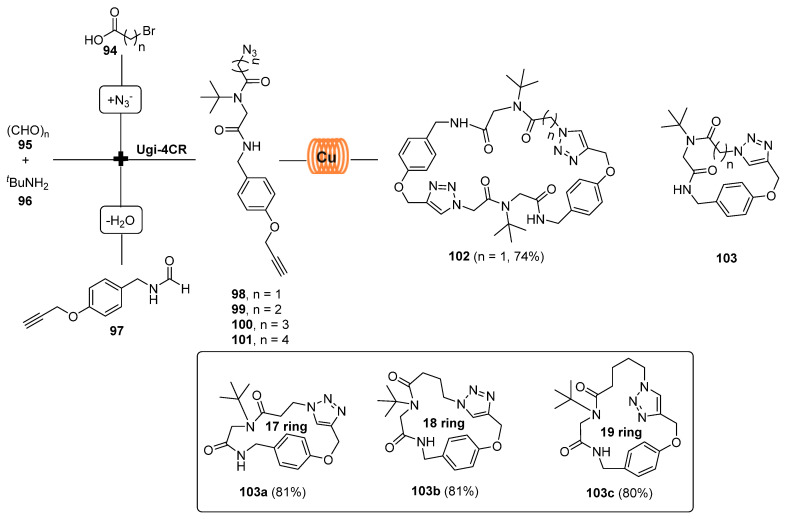
Synthesis of cyclic peptoids via flow chemistry approach.

A flow chemistry approach for the synthesis of macrocyclic peptoids was reported by Kappe and co-workers (Scheme 24) [44]. The peptidomimetic core incorporating the alkyne and azide functionalities for IAAC was generated by Ugi-4CR. The authors brought some innovation into the flow process for the Ugi reaction by using propagylated formamide **97** from which the isocyanide was generated in situ through dehydration using the Burgess reagent. In addition, the azide moiety was also synthesized in flow through nucleophilic substitution of corresponding bromide precursor **94** with tetrabutylammonium azide. The linear peptidomimetic cores **98**–**101** were synthesized in good to excellent yields by combining all these steps in a continuous flow within a reaction time of 25 min. The Ugi adduct was then subjected to IAAC by passing through a coil reactor made of copper and, most importantly, without any additional additive. It was found that either a dimeric or a monomeric peptide was formed from the Cu-catalysed azide-alkyne cycloaddition depending on the ring strain. In the case of the Ugi adduct **98** (n = 1), the dimeric peptoid was formed, and for adducts **99** (n = 2), **100** (n = 3) and **101** (n = 4), the monomeric cyclic analogues **103a**, **103b** and **103c** were formed preferentially. With this approach, the authors could synthesize macrocyclic peptidomimetics **103a**–**c** (monomeric) of ring sizes varying from 17 to 20. 

**Scheme 25:**
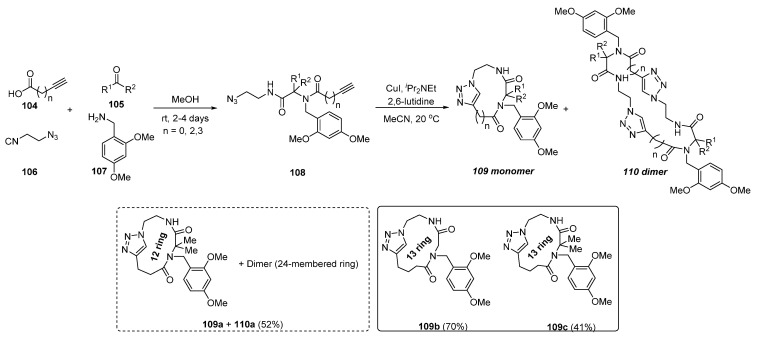
Synthesis of triazole-linked macrocyclic peptidomimetics.

In 2019, one more report came from Nenajdenko and team on the synthesis of macrocyclic (12 and 13 membered rings) peptidomimetics via the sequential Ugi-IAAC approach (Scheme 25) [45]. The authors used aliphatic and aromatic carboxylic acids bearing alkyne moiety **104**, carbonyl compounds **105**, azidoisocyanides **106** and 2,4-dimethoxybenzylamine for the MCR. Here, 2,4-Dimethoxybenzylamine **107** was used as the amine component as this could be cleaved off easily after the indented transformations. At first, the IAAC of Ugi adducts **108** derived from aliphatic carboxylic acids **104** were investigated, bearing alkyne functionality with different chain lengths. This allowed them to prepare the macrocyclic (12-membered ring) peptidomimetics as a mixture (**109a** + **110a**) with the dimer while the 13-membered macrocyclic analogues **109b** and **109c** were isolated as the sole product. 

**Scheme 26:**
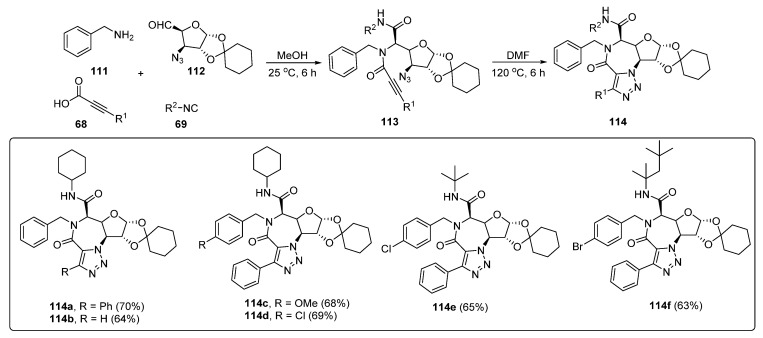
Synthesis of sugar-fused triazolodiazepines.

Reddy and co-workers utilized a sugar azidoaldehyde in an Ugi reaction for accessing sugar-fused triazolodiazepines **114** [46]. The Ugi adduct **113** was made by the MCR of substituted benzylamine **111**, sugar azidoaldehyde **112**, propiolic acid **68** and alkyl isocyanide **69** in methanol at room temperature. The resulting adduct was then subjected to IAAC through heating at 120 °C in DMF, from which the sugar fused triazolo[1,5-*a*][1,4]diazepine **114** was isolated (Scheme 26). The generality of this approach was checked by utilizing terminal and internal alkynes, differently substituted benzylamines and different alkyl isonitriles.

**Scheme 27:**
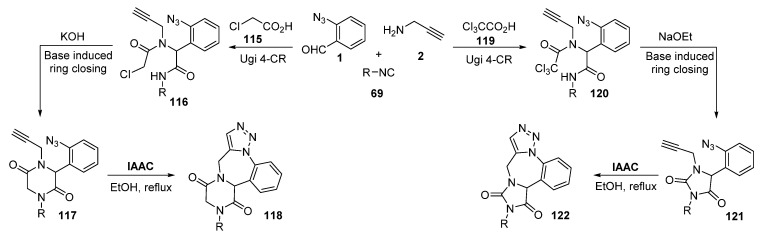
Synthesis of triazolobenzodiazepine-fused diketopiperazines and hydantoins.

Inspired by the interesting bioactivities of benzodiazepine, piperazine and hydantoin motifs, we reported an approach for the synthesis of triazolobenzodiazepine-fused diketopiperazines **118** and hydantoins **122** [47]. These tetracyclic compounds were accessed by following a three-step protocol involving an Ugi reaction, a base-induced ring closing and an IAAC. The synthesis of triazolobenzodiazepine-fused diketopiperazine **118** started with the Ugi reaction involving o-azidobenzaldehyde **1**, propargylamine **2**, an isocyanide and 2-chloroacetic acid **115** (Scheme 27). The Ugi adduct **114** was then subjected to base-induced ring closing via sonication to afford diketopiperazine moiety **117** which, on subsequent IAAC, afforded the expected tetracyclic scaffold **118**. Fused hydantoin derivatives **122** were prepared by starting with trichloroacetic acid **119** instead of 2-chloroacetic acid for the Ugi MCR. We have found that all these three steps could be performed in one-pot triazolobenzodiazepine-fused diketopiperazines in satisfactory overall yields. The drawbacks of our methodology were the narrow substrate scope with respect to o-azido-aryl/heteroaryl-aldehydes and the limited variations that could be introduced to the triazolobenzodiazepine-fused hydantoin motifs.

**Scheme 28:**
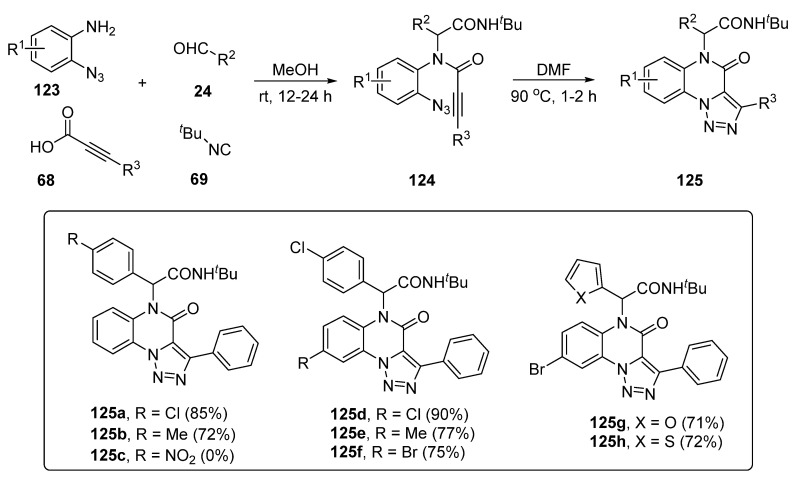
Synthesis of 1,2,3-triazolo[1,5-*a*]quinoxalin-4(5*H*)-ones.

Ding and co-workers recently reported a sequential approach for the synthesis of 1,2,3-triazolo[1,5-*a*]quinoxalin-4(5*H*)-ones (Scheme 28) [48]. The first step of the sequential process was an Ugi reaction involving 2-azidobenzenamines **123**, aldehydes, propiolic acids and isocyanides which furnished the intermediate **124** for intramolecular dipolar cycloaddition. The Ugi adducts, upon heating to 90 °C in DMF, afforded 1,2,3-triazolo[1,5-*a*}quinoxalin-4(5*H*)-ones **125** in good yields via IAAC. The authors observed the formation of products in good yields while using 2-azidobenzenamines with electron-donating group substituents. A slight decrease in the yields of 1,2,3-triazolo[1,5-*a*]quinoxalin-4(5*H*)-ones were detected when halogens were present on 2-azidobenzenamines. The reaction with 4-nitrobenzaldehyde failed to afford any product which is contrary to what is expected as Ugi reaction is expected to work well with electron deficient aldehydes.

## 4. Concluding Remarks

In this review, we have covered reports describing a sequential MCR-IAAC approach for the generation of 1,2,3-triazole-fused heterocycles. As one can see, all the methodologies were cleverly designed to place the azide and alkyne functionalities appropriately after the MCR. Most of the MCR adducts, when subjected to IAAC, did not require Cu-catalysis to facilitate the dipolar cycloaddition leading to medium-sized rings (ring size = 5–8). On the other hand, whenever macrorings are to be formed by incorporating 1,4-disubstituted rather than 1,5-disubstituted triazoles, the CuAAC protocol was preferred. In most of the reports, at least one component (of the MCR) was having two functional groups—for example, azide and aldehyde as in *o*-azido benzaldehyde or amine and alkyne as in propargyl amine. This idea of placing two functionalities in one component generates the MCR adduct having the azide and alkyne functionalities positioned in such a way that a facile IAAC is possible. There are more reports on 4-CR in comparison with the 3-CR strategies. This might be due to the availability of well-established four component MCRs and the respective starting materials. By combining MCR and IAAC in sequence, a plethora of triazole-fused heterocycles could be generated. Most importantly, the starting materials for these MCRs could be accessed and varied with ease.

There are immense possibilities for the design of new MCRs (with a possibility for subsequent IAAC) leading to interesting heterocyclic scaffolds that can form potential drug candidates. However, until now, none of the compounds that have been reviewed here were tested for biological activity, despite being attractive molecules. For example, 1,2,3-triazole-fused benzodiazepines mentioned in this review are structurally close to benzodiazepine drug molecules such as triazolam. Another important application that can be devised with the sequential MCR-IAAC approach is in the generation of novel triazole-linked peptidomimetics. This topic of review will be interesting to organic chemists and medicinal chemists, and we hope that the scientific community will be stimulated to devise new combinations of MCR and IAAC since many additional possibilities exist, which may be targeted for important applications.

## Data Availability

No new data were created or analyzed in this study. Data sharing is not applicable to this article.
